# Isolation and characterization of centromeric repetitive DNA sequences in *Saccharum spontaneum*

**DOI:** 10.1038/srep41659

**Published:** 2017-01-30

**Authors:** Wenpan Zhang, Sheng Zuo, Zhanjie Li, Zhuang Meng, Jinlei Han, Junqi Song, Yong-Bao Pan, Kai Wang

**Affiliations:** 1 Center for Genomics and Biotechnology, Fujian Provincial Key laboratory of Haixia applied plant systems biology, Haixia Institute of Science and Technology, Fujian Agriculture and Forestry University, Fuzhou, Fujian 350002, China; 2 Key Laboratory of Education Ministry for Genetics, Breeding and Multiple Utilization of Crops, Fujian Agriculture and Forestry University, Fuzhou, Fujian 350002, China; 3 College of Life Science, Fujian Agriculture and Forestry University, Fuzhou, Fujian 350002, China; 4 Texas A&M AgriLife Research Center at Dallas, Dallas, TX 75252, USA; 5 USDA-ARS, Sugarcane Research Unit, Houma, LA 70360, USA; 6 National Engineering Research Center of Sugarcane, Fujian Agriculture and Forestry University, Fuzhou, 350002, China

## Abstract

Sugarcane (*Saccharum* hybrids *spp*.) is the most important sugar crop that accounts for ~75% of the world’s sugar production. Recently, a whole-genome sequencing project was launched on the wild species *S. spontaneum*. To obtain information on the DNA composition of the repeat-enriched region of the centromere, we conducted a genome-wide analysis of the DNA sequences associated with CenH3 (a mutant of histone H3 located in eukaryote centromeres) using chromatin immunoprecipitation followed by sequencing (ChIP-seq) method. We demonstrate that the centromeres contain mainly SCEN-like single satellite repeat (Ss1) and several Ty3/*gypsy* retrotransposon-related repeats (Ss166, Ss51, and Ss68). Ss1 dominates in the centromeric regions and spans up to 500 kb. In contrast, the Ty3/*gypsy* retrotransposon-related repeats are either clustered spanning over a short range, or dispersed in the centromere regions. Interestingly, Ss1 exhibits a chromosome-specific enrichment in the wild species *S. spontaneum* and *S. robustum*, but not in the domesticated species *S. officinarum* and modern sugarcane cultivars. This finding suggests an autopolyploid genome identity of *S. spontaneum* with a high level of homology among its eight sub-genomes. We also conducted a genome-wide survey of the repetitive DNAs in *S. spontaneum* following a similarity-based sequence clustering strategy. These results provide insight into the composition of sugarcane genome as well as the genome assembly of *S. spontaneum*.

The centromere is a chromosomal domain that directs the assembly of kinetochore, which mediates chromosome segregation by interacting with spindle microtubules. A typical feature for centromeric chromatin is the presence of CenH3 (CENP-A in mammals), which is a mutant of histone H3. Studies have revealed that CenH3 present in all eukaryote centromeres studied^1^,^2^,^3^,^4^. Thus, centromeric chromatin is defined by the presence of CenH3. Centromeric DNA is composed of satellite DNAs and highly repeated centromeric retrotransposons (CRs). Centromeric satellite DNAs are usually mega base-sized arrays with a monomer size ranging from 100–200 to thousands of base pairs^5^,^6^,^7^. In humans, satellite DNAs may dominate the entire functional region of the centromere^3^,^4^,^8^,^9^,^10^. CRs are also enriched in eukaryote centromeres, especially in plants^4^,^9^,^11^. In the centromeres of maize and rice, CRs are clustered, span over a long range, and are always intermingled with centromeric satellites^12^,^13^,^14^. Most identified CRs in plants belong to the Ty3/*gypsy* group of long terminal repeat (LTR) retrotransposons^11^,^15^. The most striking structural feature of a plant CR is a C-terminal chromodomain of the integrase gene, which may be responsible for centromere-specific insertion^15^. Moreover, sequence analyses have demonstrated that some centromeric satellite DNAs originated from retrotransposons^7^,^9^,^16^,^17^,^18^,^19^,^20^. Thus, the CRs may play a core role in the occurrence of classical repeat-enriched centromeres.

Although many draft genomes of variant species have been reported in recent decades, whole centromere DNA sequencing, even the fine-scale genetic and physical mapping of centromeres, remains a challenge because of highly repetitive nature of centromeric DNA. The chromatin immunoprecipitation followed by sequencing (ChIP-seq) method allowed us to isolate the DNA sequences associated with CenH3 (CENP-A in mammals), which is a mutant of histone H3 and presents in all eukaryote centromeres studied^1^,^2^,^3^,^4^. In plants, extensive studies have been conducted to analyze the DNA composition of CenH3-associated centromeric chromatin using ChIP-seq^6^,^13^,^19^,^21^,^22^,^23^,^24^,^25^,^26^,^27^. However, poorly assembled reference centromeres often hamper the application of ChIP-seq in the characterization of centromeric DNAs. Recently, an alternative approach based on the evaluation of the enrichments of clustered repeats from the whole genome was used in centromeric DNA studies^7^,^28^,^29^,^30^. In this method, a reference repeat sequence from the whole genome was first generated and then clustered by evaluating mutual similarities between sequences to identify groups of densely connected reads^28^,^29^,^30^. Then, the potential centromeric repeat clusters were identified by evaluating the relative enrichments of the ChIP-seq reads with respect to the reference control. Several studies conducted on plants using this approach have demonstrated that it is an efficient method to assay the centromeric repeat DNA compositions of various species, especially those without a genome assembly or poorly assembled centromere regions^7^,^31^,^32^,^33^.

Sugarcane (*Saccharum* hybrids *spp*.) is the most important sugar crop that accounts for ~75% of the world’s sugar production^34^. The *Saccharum* genus is composed of two wild species, i.e., *S. robustum* and *S. spontaneum*, and four groups of formerly cultivated clones: *S. officinarum, S. barberi, S. sinense*, and *S. edule*. Both wild species contain large amounts of natural genetic variations and have a wide range of chromosome numbers and ploidy levels with 2*n* = 6*x* − 8*x* = 60–170 for *S. robustum* and 2*n* = 32–128 for *S. spontaneum*^35^,^36^,^37^. All modern sugarcane cultivars are derived from interspecific crosses between *S. officinarum* and *S. spontaneum*^38^,^39^. The wild clones of *S. spontaneum* were used to introduce disease resistance, vigor, stubbling, and other traits into *S. officinarum* and thus, a series of backcrosses with *S. officinarum* were conducted to restore the high sugar content trait, a process called Noblization (Roach 1969). The resulting modern sugarcane cultivars (*Saccharum* spp. hybrids) are highly polyploid interspecific hybrids and represent the most genetically complex crop ever studied^39^. Estimated monoploid sugarcane genome size is approximately 930 Mb, which is similar to the genome size of sorghum, closely related grass^34^. Thus, the genome of sugarcane cultivars will have a DNA content of about 10 Gb^34^. Recently, a whole-genome sequencing project was launched on a *S. spontaneum* clone SES208 (2*n* = 8*x* = 64). However, the octaploid complexity of its nuclear genome, especially the high-level content of repetitive sequences (approximately 60%) has hindered severely the assembly process of the whole genome (Ray Ming, personal communication). In this study, we conducted a genome-wide assay of centromeric repetitive DNA sequence repeats on SES208 following CenH3-based ChIP-seq approach^28^,^29^,^30^. The isolated centromeric repeats were confirmed by cytological analyses. We revealed the origin, structure and distribution of each repeat in the centromeres of SES208. These results will contribute to our understanding of sugarcane centromeres and also facilitate sugarcane whole-genome sequencing.

## Results

### Composition of the repetitive DNAs in *S. spontaneum* clone SES208

**Figure 1:**
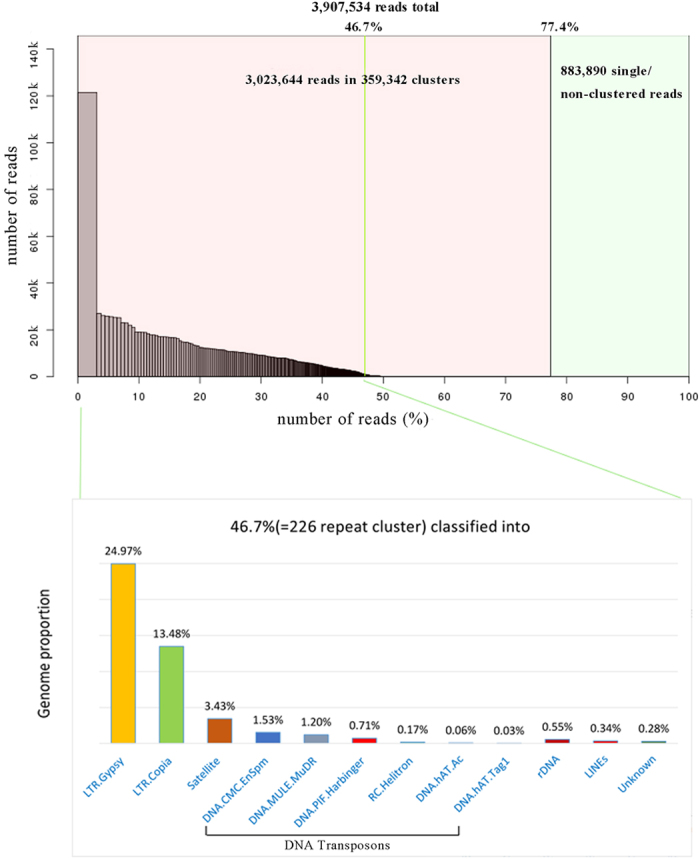
The composition and annotation of repetitive DNAs in *S. spontaneum* clone SES208. (**A**) Summary of the contents of the repeat family and single copy reads. A total of 359, 342 repeat clusters were generated, among which 226 repeat clusters (accounting for 46.7% of the genome) were analyzed further. (**B**). Annotation and the genome proportions of the 226 repeat clusters.

To provide a reference repeat database for the analysis of the centromere repetitive sequences, the input DNA was sequenced using HiSeq 2500 platform. A total of 75.9 million 100-bp pair-end reads were obtained. Of which, 3.9 million reads were randomly selected to generate repeat clusters using the RepeatExplorer software^30^. This analysis resulted in a total of 359, 342 repeat clusters and 883, 890 single/non-clustered reads. The 359, 342 clusters represented different repeat families in SES208 genome that accounted for 77.4% of the analyzed 3.9 million reads. Among these clusters, 226 clusters that accounted for 46.7% of the genomic reads were relatively enriched in the SES208 genome (genome proportion>0.01%) (Fig. 1). Thus, these 226 most highly repetitive clusters were annotated to characterize the most repeat families.

The LTR retrotransposons were the most abundant repeat families, accounting for 38.45% of the SES208 genome (Fig. 1). Among them, the Ty3/*gypsy* retrotransposons were the most enriched, representing 24.97% of the genome, followed by LTR/*copia*, accounting for 13.48% of the genome. Satellite repeats and six DNA transposons (DNA/CMC-EnSpm, DNA/MULE MuDR, DNA/PIF-Harbinger, RC/Helitron, DNA/hAT-Ac, and DNA/hAT-Tag1) were also found in the genome, representing 3.43% and 3.70% of the genome, respectively. Among the six DNA transposons, only DNA/CMC-EnSpm and DNA/MULE MuDR represented relatively high genome proportions (1.53% and 1.20%, respectively), other four DNA transposons showed less than 1% genome proportions. The rest of the repeat families, including ribosome DNA, long-interspersed nuclear elements (LINEs), and uncharacterized repeats, also showed a relatively low genome proportion of <1%.

### Computational isolation of the centromere-specific repeats in *S. spontaneum* clone SES208

**Figure 2:**
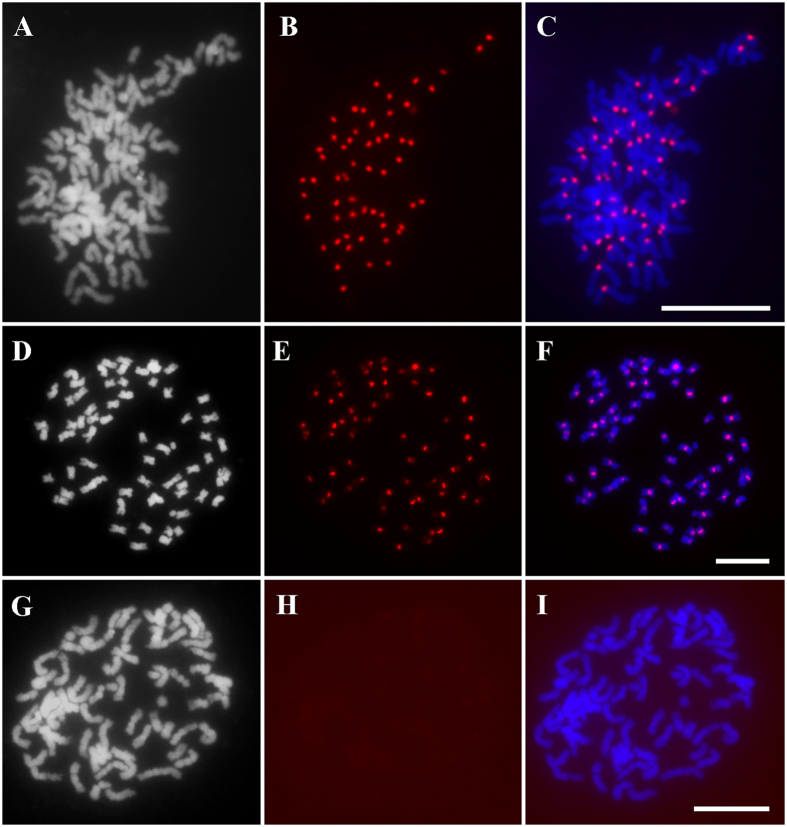
Immunoassay on rice CenH3 binding-specificity and FISH analyses of CenH3 ChIP DNA enrichment and localizations. (**A**) Metaphase chromosome spread from a *S. spontaneum* SES208 root tip cell. (**B**) Immunofluorescence signals detected at the primary chromosome constriction. (**C**) Merged image of (**A**,**B**). (**D**) Metaphase chromosome spread from a *S. spontaneum* SES208 root tip cell. (**E**) FISH signals of CenH3 ChIP DNA probe (**F**) Merged image of (**D**,**E**). (**G**) Metaphase chromosome spread from a *S. spontaneum* SES208 root tip cell. (**H**) FISH signals of mock DNA probe.(**I**) Merged image of (**G**,**H**). Bar = 10 μm.

Our immunostaining assay confirmed that the rice CenH3 antibody could also specifically recognize the CenH3 of SES208 (Fig. 2A–C). We then conducted CenH3 ChIP on *S. spontaneum* SES208. To evaluate the enrichment of centromeric DNA in our ChIP DNA, we labeled the ChIP DNA and conducted FISH analysis. We detected highly enriched signals from the centromeres of SES208, but the signals were not evident with the negative control DNA sample under the same conditions (mock, see Methods) (Fig. 2D–I).

**Figure 3:**
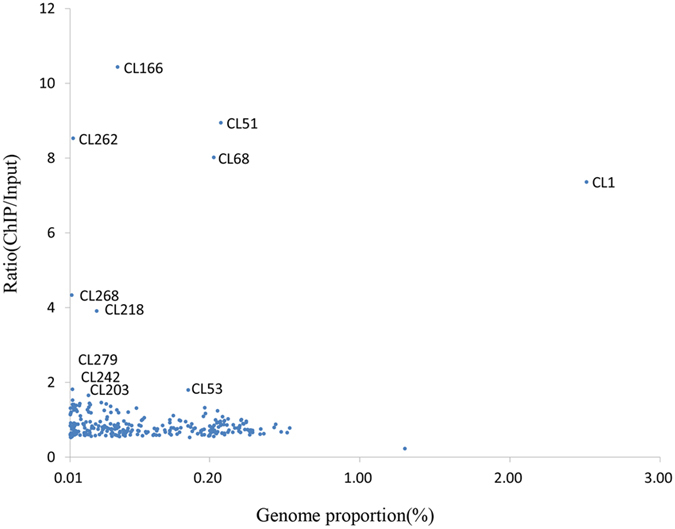
Relative enrichments of repeat DNA families in the ChIP-seq data and SES208 genome. Repeat clusters are represented by dots. The x-axis is the genome proportion for each cluster. The y-axis is the ratio of the ChIP-seq reads to input-seq reads, representing the enrichment of each corresponding cluster from the ChIP-seq data.

A total of 73.2 million 100-bp pair-end sequence reads from ChIP DNA were obtained. These CenH3 ChIP DNA reads and the 75.9 million input DNA reads were mapped to all repeat clusters using BLAST (e-value, 1e-8). The read number ratios of ChIP DNA relative to the input DNA, which indicated the level of enrichment of each repeat family in the centromere, were calculated for all clusters (Fig. 3). The sequence proportion of each repeat family was estimated based on the number of sequence reads associated with individual clusters. Seven repeat clusters showed obvious higher ratios (>2) than the others, suggesting that these seven clusters were most likely the centromeric DNAs. These seven centromeric repeat clusters having a ChIP/input ratio >2 represented a total of 3.14% of the genome proportion. Only the centromeric repeat cluster 1 showed a relatively high genome proportion (2.5%), all other six repeat clusters had a very low genome proportion (from 0.046% for cluster 218 to 0.260% for cluster 51) (Fig. 3, Table 1), indicating that the repeat elements in these six clusters had relatively low copy numbers when compared with those of repeat cluster 1 in the SES208 genome.

### Cytological confirmation of computationally identified centromeric repeats

**Figure 4:**
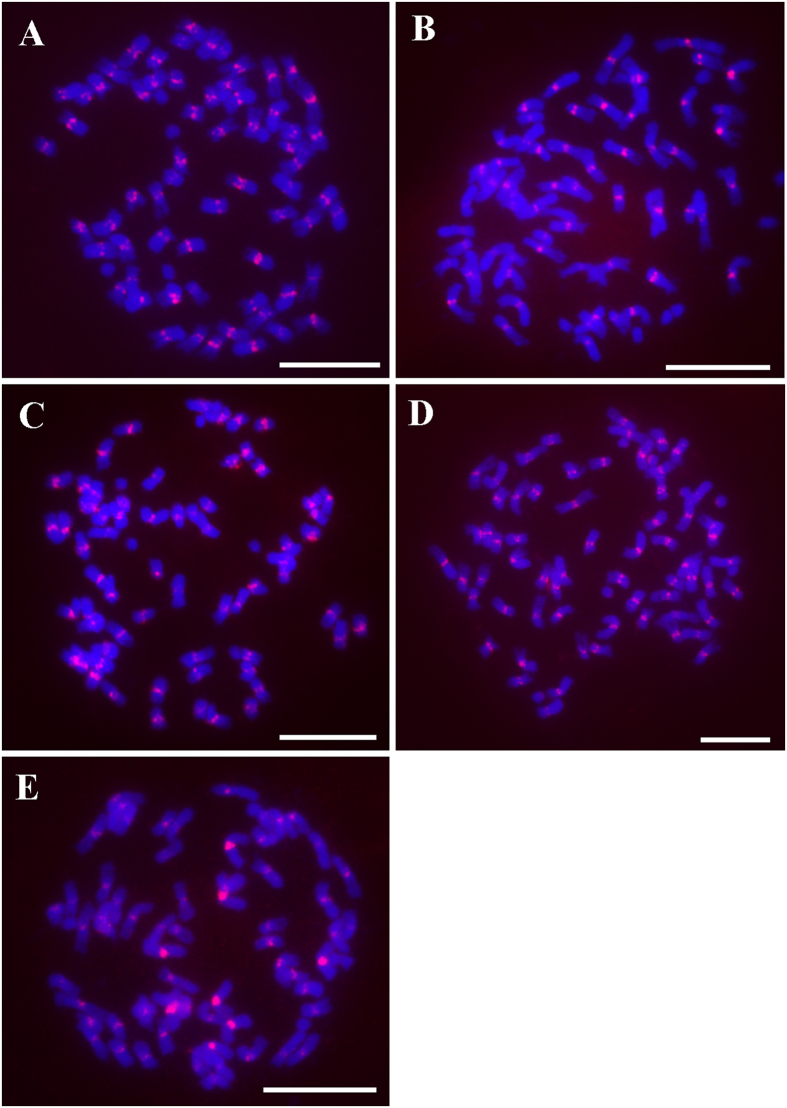
FISH mapping of centromeric repeats in *S. spontaneum* clone SES208. Five repeats, namely, Ss166 (**A**), Ss51 (**B**), Ss262 (**C**), Ss68 (**D**), and Ss1 (**E**), were mapped to the metaphase chromosomes. Centromere-specific FISH signals were detected from these five repeat probes. Bar = 10 μm.

One contig with the highest read depth for each of the seven repeat clusters was selected as the representative. In the FISH assay, all seven repeat sequences generated strong FISH signals in the centromeres (Fig. 4 and S1), confirming that these repeat sequences were components of centromeric DNAs. Five of these seven repeats, namely, Ss1, Ss166, Ss51, Ss262, and Ss68, generated centromere-specific signals from all 64 chromosomes of SES208, indicating these repeats colonized and spread specifically in the centromeres. However, the signal intensities from different centromeres varied for individual repeats, suggesting that each centromere might have a different copy number. Interestingly, we always observed eight higher intensity FISH signals for repeat Ss1 than other centromeric repeats (Fig. 4E), suggesting an enrichment of this repeat in these eight chromosomes. For repeats Ss268 and Ss218, in addition to the centromeric FISH signals, dispersed FISH signals on chromosome arms were also detected (Figure S1), suggesting that these two repeats are not centromere-specific. Therefore, these two repeats were excluded from further centromeric analyses.

To mine the centromeric repeats at a wider scale, we also analyzed four other repeat clusters (Cl242, Cl53, Cl203, and Cl279), which had relatively high ChIP/input ratios (>1.5) (Fig. 3). However, no centromeric FISH signals were detected using these repeats, implying that these four as well as other repeat clusters with lower ChIP/input ratios are likely non-centromeric. Based on all these results, the five repeat sequences (Ss1, Ss166, Ss51, Ss262, and Ss68) that generated strong centromere-specific FISH signals were selected for further analyses.

### Sugarcane centromeres contain both satellite and retrotransposon DNAs

**Figure 5:**
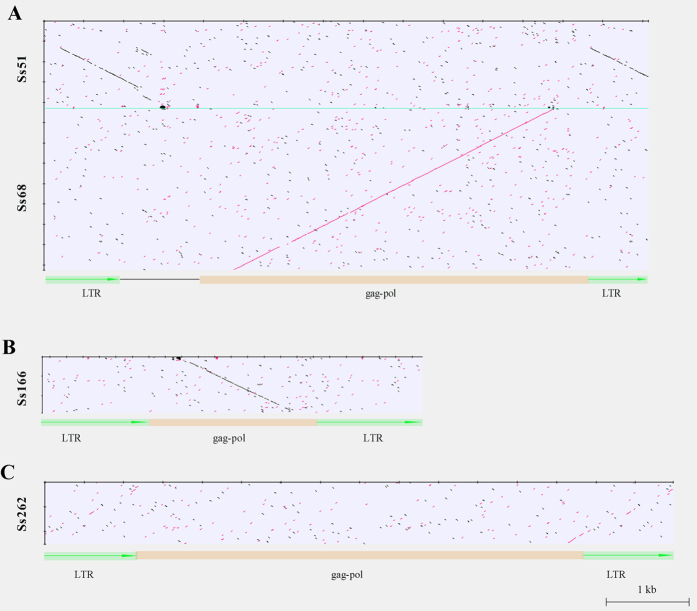
Dot-plot similarity comparisons of sugarcane centromeric repeats and other retrotransposons. Dot-plots are drawn using the Unipro UGENE software. The sequence similarities exceeding 80% over a 15-bp sliding window are displayed as black or red dots or diagonal lines. (**A**) Comparisons between the repeats Ss51 and Ss68 with a maize centromeric retrotransposon (Gene Bank: AY129008.1). (**B**) Comparisons between the repeats Ss166 with a maize centromeric retrotransposon (Gene Bank: AF078917.1). (**C**) Comparisons between the repeats Ss262 with a rice centromere retrotransposon (CRR3) (Gene Bank: DQ458292.1).

The five repeat sequences that showed centromere-specific FISH signals were blasted in the nucleotide database. Repeat Ss1 contained eight ~140-bp repetitive units, which shared high sequence similarity (74–99%) with the sugarcane centromeric satellite SCENs from an Egyptian breeding variety no. 37185^40^. For the other four repeats, varying degrees of similarities to the characterized centromeric retrotransposons were detected. Repeats Ss51 and Ss68 showed a high sequence similarity (>70%) to a centromeric retrotransposon in maize (CRM) (Gene Bank: AY129008.1), which has a full size of 7572-bp with two 931-bp long terminal repeats (LTR) (Fig. 5A). Specifically, Ss51 showed a 969-bp region of similarity to partial LTR regions (722 bp) and a 247-bp internal conjunction region of 5′ LTR (Fig. 5A). For repeat Ss68, nearly all of its sequence (97.3% or 3,855 of the full size of 3,962 bp, Table 1) was highly similar (88%) to the *gag-pol* region of the maize retrotransposon CRM (Fig. 5A). This indicated that repeats Ss51 and Ss68 may derive from different sections of one retrotransposon. The major part (84.4%, 1,225 bp) of repeat Ss166 shared 73% sequence similarity with the *gag-pol* region of another maize centromeric Ty3/*gypsy* retrotransposon (Gene Bank: AF078917.1) (Fig. 5B). For repeat Ss262, no significant similarity was found, except for a short region (~136 bp) that shared 72% sequence similarity with the *gag-pol* region of a rice centromeric retrotransposon CRR3 (Gene Bank: DQ458292.1) (Fig. 5C). Additional BLAST search in the protein database using repeats Ss51, Ss68 and Ss166 as quires revealed a large number of similarities (30–88%) with the Ty3/*gypsy* retrotransposon protein, thereby confirming their retrotransposon origin.

### The structure of centromeric repetitive DNA sequence repeats revealed by fiber-FISH

**Figure 6:**
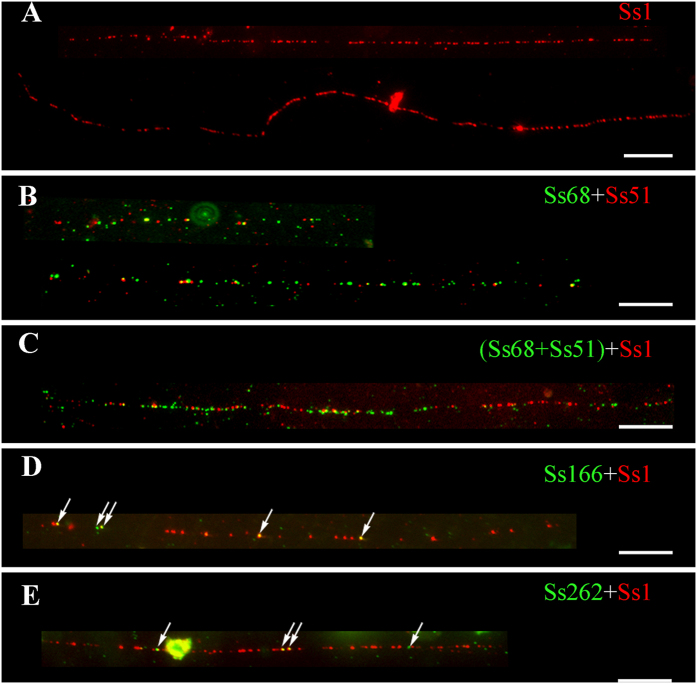
Fiber-FISH mapping of the centromeric repeats in *S. spontaneum* clone SES 208. (**A**) Two complete fiber-FISH signals derived from the satellite repeat Ss1 probe. (**B**) Two partial fiber-FISH signals derived from Ss68 (green) and Ss51 (red) probes. (**C**) One complete fiber-FISH signal derived from Ss68 + Ss51 (green) and Ss1 (red) probes. (**D**) One complete fiber-FISH signal derived from Ss166 (green) and Ss1 (red) probes. The signal spots of Ss166 probe are indicated by arrows. (**E**) One complete fiber-FISH signal derived from Ss 262 (green) and Ss1 (red) probes. The signal spots of Ss262 probe are indicated by arrows. Bar = 10 μm.

As expected, long contiguous fiber-FISH signals were observed with the repeat Ss1 probe (Fig. 6A), confirming its tandem repeat structure. We selected the ten longest fiber-FISH signals for length measurements. The length varied from 132.0 to 181.4 μm, with an average of 153.5 μm (Table S2), suggesting that the centromeric repeat Ss1 could span up to ~400 to 500 kb in *S. spontaneum* clone SES208 (1 μm = 3.24 kb)^41^.

Continuous signal spots were also detected when using repeats Ss51 and Ss68. Interestingly, fiber-FISH signals from Ss51 and Ss68 were consistently associated with the same DNA fibers (Fig. 6B) in our dual-color fiber-FISH experiment, confirming that repeats Ss51 and Ss68 represented different parts of the same repeat unit (one retrotransposon). However, the majority of the signals from this retrotransposon were short, spanning an average of 101 kb (Table S2, *n* = 19) in the genome. Moreover, the densities of the fiber-FISH signal spots of these two probes were consistently lower than those of Ss1 (Fig. 6A and B), suggesting that there were other sequence(s) inserted within this retrotransposon array. To find out the relationship between the centromeric satellite repeat Ss1 and the CR represented by repeat Ss51 and Ss68, dual-color fiber-FISH experiments were also conducted using labeled probes of Ss1, Ss51 and Ss68 (Fig. 6C). Among the sixteen fibers obtained, eight showed overlapped or partial overlapped signals (Fig. 6C). For other eight fibers, six showed only the signal of repeat Ss1 probe, while two showed signals of the CR probe, either Ss51, or Ss68, or both probes that were labeled with the same color (Fig. 6C). The results indicated that this highly repeated retrotransposon was intermingled with satellite repeat Ss1 in some centromeres of the SES208 genome.

No continuous fiber-FISH signal spots were found for Ss166 and Ss262 probes. In dual-color fiber-FISH using these two probes with repeat Ss1, we detected dispersed signal spots that overlapped with the signals of repeat Ss1 (Fig. 6D and E), suggesting that both repeats Ss166 and Ss262 were dispersed in centromeres.

### Enrichment of the SCEN-like satellite repeat was chromosome-specific in sugarcane

**Figure 7:**
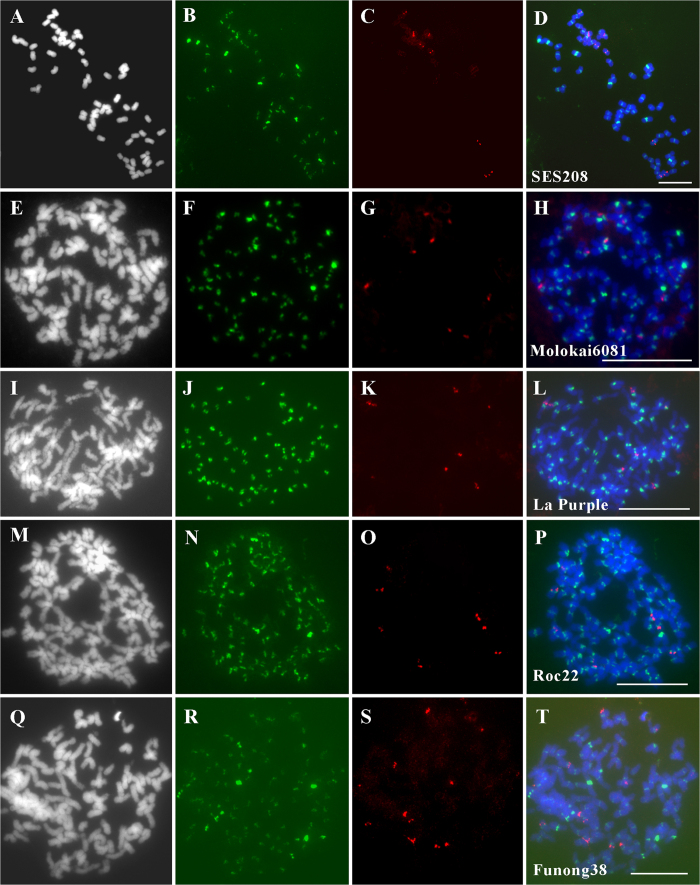
FISH assay of the satellite repeat Ss1 in other sugarcane species. The probes of repeat Ss1 and 5S rDNA were hybridized simultaneously to the metaphase chromosomes of *S. spontaneum* clone SES208 (**A**–**D**), *S. robustum* clone Molokai6081 (**E**–**H**), *S. officinarum* clone LA Purple (**I**–**L**) and two sugarcane cultivars, ROC22 (**M**–**P**) and Funong38 (**Q**–**T**). Bar = 10 μm.

An interesting finding of the FISH assay was that we consistently observed eight centromeres showing much stronger FISH signals with the repeat Ss1 probe than other probes (Fig. 4E), suggesting that these eight centromeres contain significantly more copies of repeat Ss1. Previous studies have demonstrated a high similarity within the eight su-bgenomes (chromosome set of a monoploid) for *S. spontaneum* SES208 and suggested its auto-octaploid identity of 2*n* = 8*x* = 64^34^,^36^. Thus, it is most likely that there are eight homologous chromosomes for one specific chromosome (designated as the Chromosome Ss1) distribution among the eight sub-genomes. However, a recent study revealed that rapidly evolving centromeric DNAs could colonize and proliferate in the centromeres of chromosomes belonging to one sub-genome after polyploidy formation^33^. Thus, we cannot rule out the possibility that these eight chromosomes also belong to one sub-genome. To clarify this, we conducted a FISH assay using both repeat Ss1 and 5 S rDNA probes (Fig. 7A–D). The results showed that there were eight chromosomes bearing the 5S rDNA signals (Fig. 7C), and those chromosomes are theoretically the eight homologous chromosomes derived from one specific chromosome (designated as the Chromosome 5S) in this octaploid sugarcane. If these eight chromosomes bearing the brighter Ss1 signals were a complete chromosome set of one sub-genome, we would have seen one chromosome bearing FISH signals from both Ss1 and 5S rDNA. However, this was not the case in this study (Fig. 7A–D), indicating that the eight chromosomes with brighter Ss1 signals were not members of one sub-genome. Furthermore, the morphologies of these eight chromosomes were not significantly different (Figure S2). All these results suggested that the eight chromosomes bearing brighter Ss1 signals were homologous to one another and distributed among the eight sub-genomes.

Additional FISH analyses were also conducted on another wild species *S. robustum* (clone Molokai6081), *S. officinarum* (clone LA Purple), and two modern sugarcane cultivars (ROC22 and Funong38). Significantly brighter FISH signals of repeat Ss1 were also detected in one set of eight chromosomes in *S. robustum* (Fig. 7F), which did not bear the 5S rDNA signals either (Fig. 7G). However, the signal intensities of the Ss1 satellite repeat showed a graduated difference, and obviously brighter Ss1 signals were hardly seen in *S. officinarum* (Fig. 7I–L) and cultivars ROC22 and Funong38 (Fig. 7Q–T). As *S. officinarum* is domesticated from wild species *S. robustum*, and cultivars are the interspecific hybrids of *S. officinarum* and wild species *S. spontaneum*^37^, it is concluded that the SCEN-like satellites which has a equal chromosome-Ss1 enrichments in *S. robustum* and *S. spontaneum* may have undergone uninformed deletion or proliferation in different centromeres during domestication. Interestingly, SCEN-like satellites have equal chromosome-Ss1 enrichment patterns in *S. robustum* and *S. spontaneum*.

## Discussion

In eukaryotes, a significant fraction of the genome is comprised of repetitive DNAs, the component of which is often greater than the coding sequence component and is also referred to as the “dark matter” of the genome^42^. Researchers showed that the repetitive DNAs play a role in numerous cell processes^43^,^44^. Therefore, understanding the contents and origins of repetitive DNAs represents an important step towards completely deciphering the organization and function of the genome sequence^45^. However, the contents of repetitive DNAs derived from whole-genome sequencing are likely under-estimated because the highly repeated DNAs regularly fail to be assembled due to the technology barrier. For example, approximately half of the human genome was previously identified as TEs and other repeats in a previous human genome sequencing dataset^46^. However, an additional 20% or more repetitive or repeat-derived DNAs were identified using an alternative *de novo* strategy recently^45^. This phenomenon was also found in animals and plants^31^,^33^,^47^. In this study, we applied a method to assess repetitive DNA composition using similarity-based sequence clustering and annotation rather than the genome assembly. This approach has been applied effectively to evaluate the repetitive DNA composition for whole genome or centromeric regions in other plants^7^,^31^,^32^,^33^. It is notable that approximately 77% of the SES208 genome is comprised of mobile elements or other repeat structures (Fig. 1), demonstrating a high proportion of repetitive elements in the SES208 genome. We believe that this survey of the compositions and genome proportions of the repetitive families will contribute to SES208 genome sequencing and further sugarcane genome studies.

In rice, maize and sorghum, the centromeric satellite repeats can span from hundreds of Kb to several Mb^12^,^14^,^48^, which has hampered the DNA sequence assembly of the centromeric region. Our Fiber-FISH assay revealed that the repeat Ss1 could span up to 500 kb in SES208. We cannot determine if the data represent the entire size of the Ss1 arrays in one centromere because the satellite repeats can be interrupted by CRs (Fig. 6C). However, the data indicate that the DNA sequence assembly of the centromeric regions remains a challenge for SES208 because reconstruction of such long tandem arrays is not feasible, even with the latest PacBio sequencing platform (read length up to 40 kb with an average of 10–15 kb). Alternatively, cytological analyses based on chromosome-specific markers^12^ or individual chromosome addition lines^6^,^14^ might be a feasible approach to elucidate the composition and structure of centromere in sugarcane.

A common feature of typical centromeres is enriched satellites and TE repeats. Moreover, the centromeric satellites are often homogenized and thus, a single type of satellite can dominate all centromeres in most higher eukaryotes^27^. For example, the centers of centromeres are always occupied by one type of satellite repeat in major crops, such as rice (CentO)^12^, maize (CentC)^14^, sorghum (pSau3A9)^49^, *Brachypodium* (Bd_CENT)^50^, and *Arabidopsis* (pAL1)^51^,^52^. In humans, the alpha satellite dominates the entire functional region of the centromere^8^. Here, our results show that the DNA composition of the SES208 centromere is also characterized as a typical centromere. First, satellite and retrotransposon-like DNAs are the major repeat components in the centromeres (Table 1). Second, only one centromeric satellite repeat is found that accounts for 2.5% of the genome. This value is much higher than the value of TE-related or other repeats (0.57% genome proportion for the four centromere-specific repeats) (Fig. 3, Table 1). In addition, long and compact signal spots for satellite Ss1 are detected by fiber-FISH (Fig. 6A), in contrast to the short and loose signal spots for the TE-related or other repeats (Fig. 6B–E). All these data indicated that the Ss1 satellite repeat is dominantly abundant in SES208 centromeres.

Centromeric satellites evolve rapidly and can differ greatly, even among closely related species in eukaryotes^27^. However, once a satellite repeat evolves into a structure that would be favorable for the function and structure of the centromere, it will be fixed and spread to other centromeres. For example, the alpha-satellite repeat has occupied the centromeres of primate species for nearly 40 million years^19^,^53^. The results from this and several other studies have revealed that the SCEN-like satellite Ss1 colonized the centromeres of all studied clones of *S. spontaneum, S. robustum, S. officinarum*, and modern sugarcane cultivars^40^,^54^. This may suggest that SCEN-like satellite is relatively conserved or resides in the centromeres across all the species and modern sugarcane varieties of the *Saccharum* genus. Further efforts have to be devoted to verify this because the extent of diversity with regard to basic chromosome number and ploidy level is unclear in this genus.

It is known that all modern sugarcane cultivars are derived from interspecific crosses between *S. officinarum* and *S. spontaneum* conducted a century ago^38^,^39^ and *S. officinarum* was assumed to originate from *S. robustum*^35^. However, our results showed that the SCEN-like satellite repeat Ss1 exhibited chromosome-specific enrichment in *S. spontaneum* and *S. robustum*, but not in *S. officinarum* and the cultivars (Fig. 7). A recent study in maize showed that the domestication selection for centromere-like genes could cause amplification or deletion of the satellite centromere repeats in maize^26^. In cotton, the D sub-genome-derived centromeric repeats could invade the centromeres of other sub-genomes and rapidly amplify after polyploidization, which might result in indistinguishable enrichment among different centromeres for each centromeric repeat^33^. Thus, a potential explanation for our results is that the SCEN-like satellite repeat Ss1 found in *S. officinarum* and sugarcane cultivars may have undergone rapid turnover events among different centromeres during the interspecific hybridization and domestication selection process, whereas asexual vegetative propagations may have restrained the chromosome-specific enrichment pattern of the SCEN-like satellite repeat in the two wild species.

More importantly, the highly conserved pattern of the SCEN-like satellite repeat Ss1 between eight homologous chromosomes may have occurred before the polyploidization event. Another possibility is that Ss1 repeat may have undergone either uniform amplification for the eight individual chromosomes or deletion in other chromosomes. However, the latter hypothesis is unlikely because the SCEN-like satellite repeat Ss1 colonized all centromeres, and there is no evidence showing that the centromeric satellite repeat can turnover synchronously in the multiple copies of specific centromere(s) in polyploid individuals. By contrast, our results from *S. officinarum* and other cultivars suggest that either deletion or amplification of the satellite repeat Ss1 was not limited to specific centromeres (Fig. 7). Given the rapid evolution of the centromeric satellite repeat, we can anticipate that the formation of the octaploid occurs through a rapid doubling process without dramatic heterogenization between sub-genomes. This means that the *S. spontaneum* clone SES208 has an auto-octaploid genome with eight homologous sets of sub-genomes.

## Methods

### Plant Materials

Plant materials used in this study included two sugarcane cultivars, ROC22 and Funong38, and three clones of wild species, SES208 (*S. spontaneum*, 2*n* = 8*x* = 64), Molokai6081 (*S. robustum*), and LA Purple (*S. officinarum*, 2*n* = 80). All plants were grown at 30 °C in the greenhouse under natural sunlight.

### ChIP and ChIP-seq

To isolate the centromeric DNA sequences, we performed CenH3 ChIP on *S. spontaneum* SES208. A polyclonal antibody against rice CenH3 was used in this study^55^. ChIP experiments were conducted according to published protocol^55^. Young leaf tissues were collected for ChIP experiment. A fraction of micrococcal nuclease-digested genome DNAs was preserved as genomic control (designated as input DNA). Normal rabbit serum was used in the mock control. A ChIP-seq library was constructed by using NEBNext^®^Ultra™DNA Library Prep Kit (New England BioLabs Inc., Ipswich, MA, USA) according to product instructions. The library was sequenced on HiSeq 2500 (Illumina, San Diego, CA, USA) following the 100 bp paired-end genomic DNA sequencing protocol.

### Data Treatment and Centromeric Repeat Identification

The sequence reads from ChIP and input DNAs (ChIP-seq and input-seq reads, respectively) were first treated using FastUniq^56^ and Trimmomatic^57^ to remove PCR duplication and low quality reads. A two-step procedure^7^ was adopted with some modifications to determine the centromeric repetitive sequences. First, the input DNA was sequenced using HiSeq 2500 platform. A portion of randomly selected input-seq reads were used to perform graph-based clustering using the RepeatExplorer software (http://repeatexplorer.umbr.cas.cz/) with default parameters^58^,^59^. Repeats (contigs) were then identified and classified as individual repeat clusters based on their sequence similarity. Second, the ChIP-seq and input-seq reads were mapped to repeat clusters using BLASTn with E-value threshold 1e-8^60^,^60^. Reads were assigned to one cluster based on their highest similarity. The numbers of aligned reads from ChIP-seq and input-seq were counted, and the read proportion of each repeat family was calculated based on aligned read number/total read number. The ChIP/input ratios were used to evaluate the relative enrichment of repeat families in the centromere.

PCR primers were designed from bioinformatically putative repeat contigs (see Table S1). The PCR products with the corresponding sizes were recovered using a gel extraction kit (Promega, USA). Amplicons were then cloned into bacterial DH5α cells and sequenced to confirm the presence of the desired repeats. The cloned amplicons were isolated and labeled for further FISH analysis.

### Chromosomal Immunoassay

The immunoassay was performed as previously described^7^ with some modifications. Briefly, fresh root tips of SES208 were harvested and fixed in 2% (w/v) paraformaldehyde for 15 min at room temperature (RT). Root tips were washed in 1 × PBS for three times for 5 min each. A single root tip was then squashed on a glass slide with a cover slip. The cover slip was removed after freezing in liquid nitrogen, followed by dehydration in 70% ethanol for 5 min. The rice CenH3 antibody (20 μg/mL) was applied to the chromosome slides and incubated at 37 °C for 3 hr. The slides were washed in 1 × PBS at RT for three times and incubated with Alexa Fluor 594 Chicken anti-Rabbit IgG (Invitrogen, USA) at 37 °C for another hour. After three times of 5-min washing at RT in 1 × PBS, chromosomes were counterstained with 4, 6-diamidino-2-phenylindole and were examined under an Olympus BX63 fluorescence microscope (Olympus, Japan).

### Fluorescence *in situ* hybridization (FISH)

FISH and fiber-FISH were carried out according to published protocols^41^,^61^,^62^. The DNAs labeled with digoxigenin-dUTP (Roche Diagnostics, USA) and Biotin-dUTP (Roche Diagnostics, USA) were detected using rhodamine-conjugated anti-digoxigenin (Roche Diagnostics, USA) and fluorescein-conjugated avidin (Life Technologies, USA), respectively. DNAs were labeled with digoxigenin-dUTP and Biotin-dUTP for FISH analysis. Slides were examined under Olympus BX63 fluorescence microscope (Olympus, Japan). Chromosome and signal images were captured and merged using CellSens Dimension software (Olympus, Japan). Fiber-FISH was conducted to reveal the organization of the centromeric repeat sequences in SES208 genome. The fiber-FISH signals were measured and converted into kb using a 3.21-kb/μm conversion rate^41^.

## Additional Information

**Accession codes:** The CenH3 ChIP and input datasets were submitted to European Molecular Biology Laboratory-European Bioinformatics Institute (EMBL-EBI) (accession number PRJEB15858).

**How to cite this article**: Zhang, W. *et al*. Isolation and characterization of centromeric repetitive DNA sequences in *Saccharum spontaneum. Sci. Rep.* **7**, 41659; doi: 10.1038/srep41659 (2017).

**Publisher's note:** Springer Nature remains neutral with regard to jurisdictional claims in published maps and institutional affiliations.

## Supplementary Material

Supplementary Files

## Figures and Tables

**Table 1:** Characterizations of the centromeric repeats in clone SES208.

Repeat cluster	ChIP/Genome ratio	Genome proportion (%)	Repeat Contig	Contig length (bp)	Repeat Type
Cl166	10.44	0.075	Ss166	1,451	Ty3/Gypsy
Cl51	8.94	0.260	Ss51	2,170	Ty3/Gypsy
Cl262	8.53	0.014	Ss262	1,267	Unknown
Cl68	8.02	0.222	Ss68	3,962	Ty3/Gypsy
Cl1	7.36	2.509	Ss1	1,208	SCEN-like Satellite
Cl268	4.33	0.012	Ss268	582	Unknown
Cl218	3.91	0.046	Ss218	532	Unknown
