# Supplementary material for: Isolation and characterization of centromeric repetitive DNA sequences in Saccharum spontaneum
Source: Sci Rep. 2017 Jan 30;7:41659. doi: 10.1038/srep41659 (PMC5278356; doi:10.1038/srep41659)
Supplement: Supplementary Files [file srep41659-s1.pdf]

SUPPLEMENTARY INFORMATION

Isolation and characterization of centromeric repetitive DNA sequences in *Saccharum spontaneum*

Wenpan Zhang, Sheng Zuo, Zhanjie Li, Zhuang Meng, Jinlei Han, Junqi Song, Yong-Bao Pan, Kai Wang

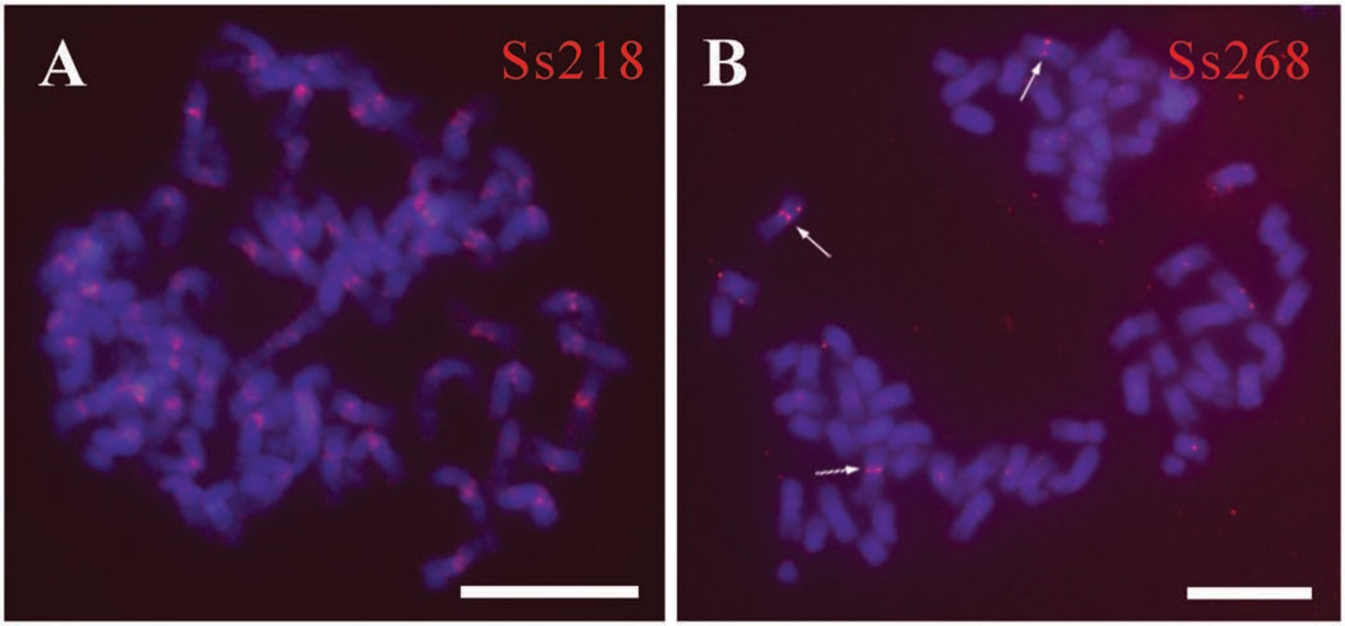

Figure S1. FISH mapping of repeats Ss218 and Ss268.

(A) Signals from Ss218 probe are concentrated in the centromeres in clone SES208. However, signals from pericentromeric or interstitial regions can also be detected. (B) Weak and dispersed FISH signals were detected from Ss268 probe. Signals located in the centromeric regions are indicated by arrows. Bar = 10 μm

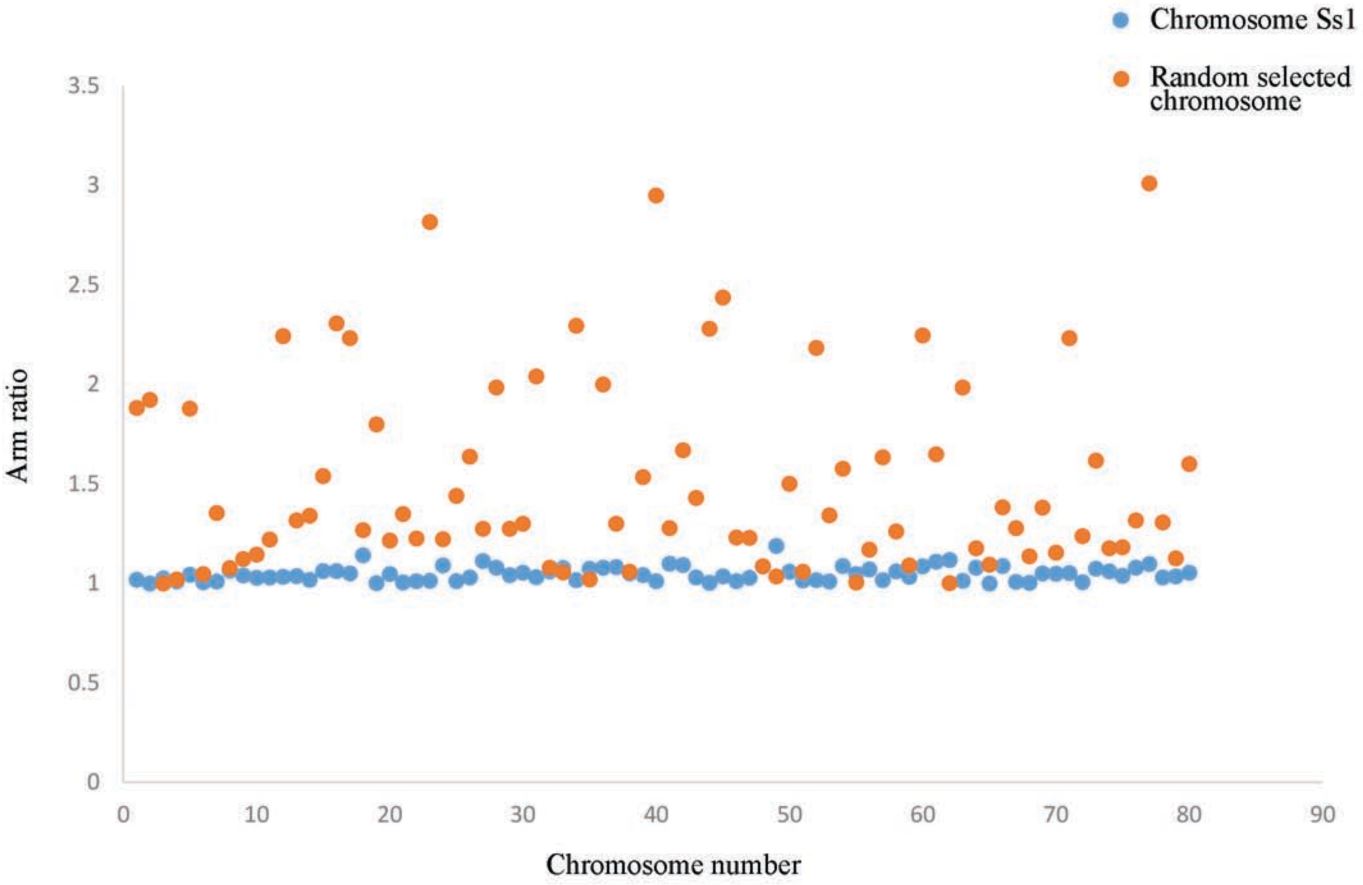

Figure S2. Arm radios of repeat Ss1 between brighter signal-bearing chromosomes and randomly selected chromosomes of clone SES208.

Arm length of the eight brighter Ss1 signal-bearing chromosomes and eight randomly selected chromosomes (n = 80) were measured, and long arm/short arm ratios were calculated and are represented by dots. The arm ratios of the brighter Ss1 signal-bearing chromosomes are almost consistent across the 80 measurements. In contrast, the arm ratios of the randomly selected chromosomes vary across a wide range. This reveals that the brighter Ss1 signal-bearing chromosomes have consistent morphology and should belong to a homologous set of eight chromosomes.

Table S1 PCR primers used in the amplification and isolation of centromeric repeats.

Repeat contig	Primer	Primer sequence (5'-3')	Length (bp)	Tm (^{o}C)	GC (%)	Amplicon size (bp)
Ss166	WL063F	TCCTTGCTTTAGCCCACCT	19	57.3	52.6	665
	WL063R	GCATTCATCCAATCCACCA	19	56.7	47.4	
Ss 51	WL067F	AGGTGGTGTATCGGTTGCTAA	21	57.4	47.6	782
	WL067R	TTGGGAAGGGTGATTCAGTAG	21	56.8	47.6	
Ss 262	WL058F	GGAAACGGAGGCTTTGATAC	20	56.5	50.0	616
	WL058R	ATACAAGGAGTTAGACGGTGCT	22	55.4	45.5	
Ss 68	WL060F	GACTAAGAGACTCACGCACA	20	50.3	50.3	697
	WL060R	CCACATCCCCATCCCTA	17	53.3	53.3	
Ss1	WL081F	CAGCTAGGAGAACCATCGAGT	21	56.2	52.4	387
	WL081R	TCAGCCCATACGGAGAAAC	19	55.6	52.6	
Ss268	WL066F	GCTCATCCAAGCAAAGTCA	19	53.9	47.4	214
	WL066R	TCTCTATGTCCTCTACTTTCTCG	23	53.1	43.5	
Ss218	WL059F	AGCCACCCTTCCCCTAT	17	53.6	58.8	185
	WL059R	CCTCATCATTCCCACCC	17	52.6	58.8	
Ss242	WL082F	CAGCACAAGTAGACCATAGC	20	50.6	50.0	181
	WL082R	ACAGGTTGAAGGGAAAATAC	20	51.0	40.0	
Ss53	WL086F	GCTGAATCGCCATAGACAAG	20	55.8	50.0	253
	WL086R	GGACAGAAATCCAACAACCC	20	56.5	50.0	
Ss203	WL092F	CCCTCTTCATCTACCAAAAC	20	51.3	45.0	483
	WL092R	ATTCCATCTAAGTGTCCGTC	20	51.2	45.0	
Ss279	WL095F	ATCGCTATTCCACCTTTTA	19	50.2	36.8	214
	WL095R	GACTACTGGCGGTTTCAT	18	49.9	50.0	

Table S2. Measurements of fiber-FISH signals from centromeric repeats.

Repeat	Fibers	Fiber length (µm)	Fiber length (kb)*
	1	181.42	587.81
	2	180.76	585.65
	3	174.12	564.15
**Ss1**	4	171.22	554.75
	5	144.42	467.93
	6	143.00	463.32
	7	139.94	453.41
	8	134.53	435.89
	9	133.21	431.61
	10	132.01	427.70
	**Average**	**153.46**	**497.22**
	1	124.03	401.86
	2	115.72	374.93
	3	76.12	246.63
	4	75.88	245.85
	5	39.84	129.08
	6	35.83	116.09
	7	23.41	75.85
**Ss68+Ss51**	8	19.76	64.02
	9	17.36	56.25
	10	13.81	44.74
	11	9.79	31.72
	12	9.40	30.46
	13	7.04	22.81
	14	7.03	22.78
	15	5.98	19.38
	16	4.19	13.58
	17	3.26	10.56
	18	2.80	9.07
	19	1.53	4.96
	**Average**	**31.20**	**101.08**

* A conversion rate of 3.24 Kb/µM (Wang et al. 2013) was used to convert the measurements.
